# How to Preach Effectively

**Published:** 1855-05

**Authors:** 


### HOW TO PREACH EFFECTIVELY,

And with the least wear and tear of mental and physical strength.

1st. Have a thorough knowledge of your subject.

2. Be deeply impressed with its importance.

3. Open the discourse with an earnest enunciation, in concise language, of some striking truth; this will inevitably wake up attention.

4. Then plunge *in medias res*, with the fervor of a man who is speaking for the last time as to himself or as to some one or more hearers as to him, and upon whose skirts hangs the blood of immortal souls.

5. As soon as the burden of the discourse is delivered, sit down, even if you have been speaking but twenty minutes, but fifteen, but ten! the value of a discourse is not its length, but the nailing home of some great truth on the understanding and the conscience; and be assured that such a truth is there for life. Thus you will preach easily for yourself, profitably to those who hear you.

---

The forms and ceremonies of politeness may be dispensed with, in a measure, in the relations and intimacies of one's own fireside, but kind attentions, never.

